# 

**Published:** 1863

**Authors:** 


### CONTENTS.

### ORIGINAL CONTRIBUTIONS.

ART. XIII. Physiological Incompatibility of the Respective Sexes of our Species. By W. Byrd Powell, M.D., Covington, Ky., ... 209
ART. XIV. Cold and Wet a cause of Camp Disease, and Its Modus Operandi. By C. A. Hunt, Surgeon of 126th Reg't Ill. Vols., ........ 226

### ARMY CORRESPONDENCE.

Letter from O. B. Ormsby, Assistant-Surgeon, 18th Regt. Ill. Vols., ........ 236
Letter from W. B. Witt, 1st Assistant and Acting-Surgeon, 69th Ind. Vol. Infantry ........ 240

### THE CLINIQUE.

Improved Methods of Treatment in Joint Diseases, ........ 241

### PROCEEDINGS OF SOCIETIES.

The American Medical Association, ........ 246
Esculapian Society, ........ 266

### BOOK NOTICES

Chemistry. By William Thomas Brande, D.C.L., F.R.S.L. & E. of Her Majesty's Mint, Member of the Senate of the University of London, and Honorary-Professor of Chemistry in the Royal Institution of Great Britain; and Alfred Swaine Taylor, M.D., F.R.S., Fellow of the Royal College of Physicians of London and Professor of Chemistry and Medical Jurisprudence in Guy's Hospital, ........ 270
Diseases of the Skin. By Erasmus Wilson, F.R.S. Fifth American, from the fifth and revised London edition, with plates and illustrations on wood, ........ 271
Obstetrics: The Science and Art. By Charles D. Meigs, M.D., lately Professor of Midwifery and the Diseases of Women and Children in Jefferson Medical College at Philadelphia, &c., &c., &c., &c. Fourth edition revised, with 129 Illustrations, ........ 271
Transactions of the Medical Association of Southern Central New York, at the 12th, 13th, 14th, and 15th Annual Meetings, held in 1858 '59 '60, and 61, ........ 272

### EDITORIAL.

American Medical Association, ........ 273
Calomel and Tartarized Antimony in the Army, ........ 277
Hospitality of the Profession in Chicago, ........ 278
Chancres.—By Wm. E. Bowman, M.D.—Treatment of Soft Chancre, ........ 279
On the Treatment of Consumption by the Hypophosphites. ........ 282
Iodide of Lime, ........ 283
Materia Medica and Hygiene in the University of Buffalo, ........ 285
Diphtheria, ........ 285
The Action of Expectoration, ........ 285
Academy of Medicine, Paris, ........ 285
Vivisection, ........ 285
Cinchona in Java, ........ 285
Rank of Naval Surgeons, ........ 286
Diminished Death-rate in the Army, ........ 286
The Deaths in London in 1862, ........ 286
Peruvian Physicians, ........ 286
A Cure for Fistula Lachrymalis, ........ 286
Amaurosis and Smoking, ........ 272
Calomel and Tartar Emetic, ........ 272

